# Vascularized lymph node transplantation successfully reverses lymphedema and maintains immunity in a rat lymphedema model

**DOI:** 10.1002/btm2.10301

**Published:** 2022-02-26

**Authors:** Ahmet Hamdi Sakarya, Chi‐Wei Huang, Chin‐Yu Yang, Hui‐Yi Hsiao, Frank Chun‐Shin Chang, Jung‐Ju Huang

**Affiliations:** ^1^ Division of Reconstructive Microsurgery, Department of Plastic and Reconstructive Surgery Chang Gung Memorial Hospital, Linkou Medical Center Taoyuan Taiwan; ^2^ College of Medicine Chang Gung University Taoyuan Taiwan; ^3^ Center for Tissue Engineering Chang Gung Memorial Hospital, Linkou Medical Center Taoyuan Taiwan; ^4^ Division of Craniofacial Surgery, Department of Plastic and Reconstructive Surgery Chang Gung Memorial Hospital, Linkou Medical Center Taoyuan Taiwan

**Keywords:** immunity, lymphedema, rat lymphedema model, vascularized lymph node transfer

## Abstract

Vascularized lymph node transplantation (VLNT) has shown inspiring results for the treatment of lymphedema. Nevertheless, it remains unclear how VLNT restores lymphatic drainage and whether or not immunity recovers after surgery. Hindlimb lymphedema model was created using rats with extensive groin and popliteal lymph node removable following with radiotherapy, and the lymphedema was confirmed using indocyanine green (ICG) lymphangiography and micro‐computer tomography for volume measurement. VLNT was performed 1 month later. Volume measurement, ICG lymphangiography, histology, and immune reaction were done 1 month after surgery. VLNT successfully reduced the volume of the lymphedema hindlimb, restored lymphatic drainage function with proven lymphatic channel, and reduced lymphedema‐related inflammation and fibrosis. It promotes lymphangiogenesis shown from ICG lymphangiography, histology, and enhanced lymphangiogenesis gene expression. Dendritic cell trafficking via the regenerated lymphatic channels was successfully restored, and maintained systemic immune response was proved using dinitrofluorobenzene sensitization and challenge. VLNT effectively reduces lymphedema and promotes lymphatic regeneration in the capillary lymphatic but not the collecting lymphatic vessels. Along with the re‐established lymphatic system was the restoration of immune function locally and systemically. This correlated to clinical experience regarding the reduction of swelling and infection episodes after VLNT in lymphedema patients.

## 1 INTRODUCTION

Affecting more than 16%–39% of breast cancer patients and 20%–49% of gynecological cancer patients, lymphedema is one of the most debilitating complications of cancer treatments. It strongly and negatively impacts the quality of life of cancer survivors, and thus establishing a reliable treatment is urgent and crucial.^1^, ^2^, ^3^, ^4^, ^5^

The lymphatic system is considered the third circulation system throughout the body and is vital for fluid homeostasis, maintaining immune defense and dietary fat absorption in the intestines. Damage to the lymphatic channels caused by removal of the lymph nodes followed by radiotherapy often results in blockage of lymphatic continuity. This results in disruption of lymphatic fluid and immune cell trafficking, which further damages immune function. Patients often present with localized protein‐rich fluid retention, subsequent chronic inflammation and fibrosis, and susceptibility to infection.^6^ This suggests that both fluid homeostasis and immune defense are affected by lymphedema.

Vascularized lymph node transfer (VLNT) was initiated in an animal study in 1990 for the purpose of lymphedema treatment and has been popularized in recent years with multiple donor sites explored, various recipient sites used and newly developed equipment allowing for an accurate diagnosis and real‐time intraoperative guidance.^7^, ^8^, ^9^, ^10^, ^11^, ^12^, ^13^ Although promising results of VLNT have been reported by different groups, it remains unclear whether lymphangiogenesis and the recovery of immune function are established after VLNT. Instead, bypassing lymphatic drainage by diverting it into the venous system (supramicrosurgical lymphatic‐venous anastomosis [LVA]) is recommended by some reconstructive surgeons.^14^, ^15^, ^16^, ^17^ The conflicts between lymph node transfer and LVA persuaded us to explore the role of lymph nodes in surgical treatments in lymphedema, considering the potential donor site morbidities.^18^

Frequent infection is one prominent symptom of lymphedema patients besides the presence of extremity swelling and fibrosis.^19^ To understand whether VLNT can restore immune function, we applied an animal model that mirrors the clinical situation of lymphedema, which was then treated with VLNT.^20^, ^21^ The recovery of immune defense was investigated by evaluating immune cell trafficking and the systemic immune response in addition to confirming reduced swelling and lymphedema.

## 2 RESULTS

### 2.1 VLNT successfully reduced the volume of the lymphedema hind limb

**FIGURE 1:**
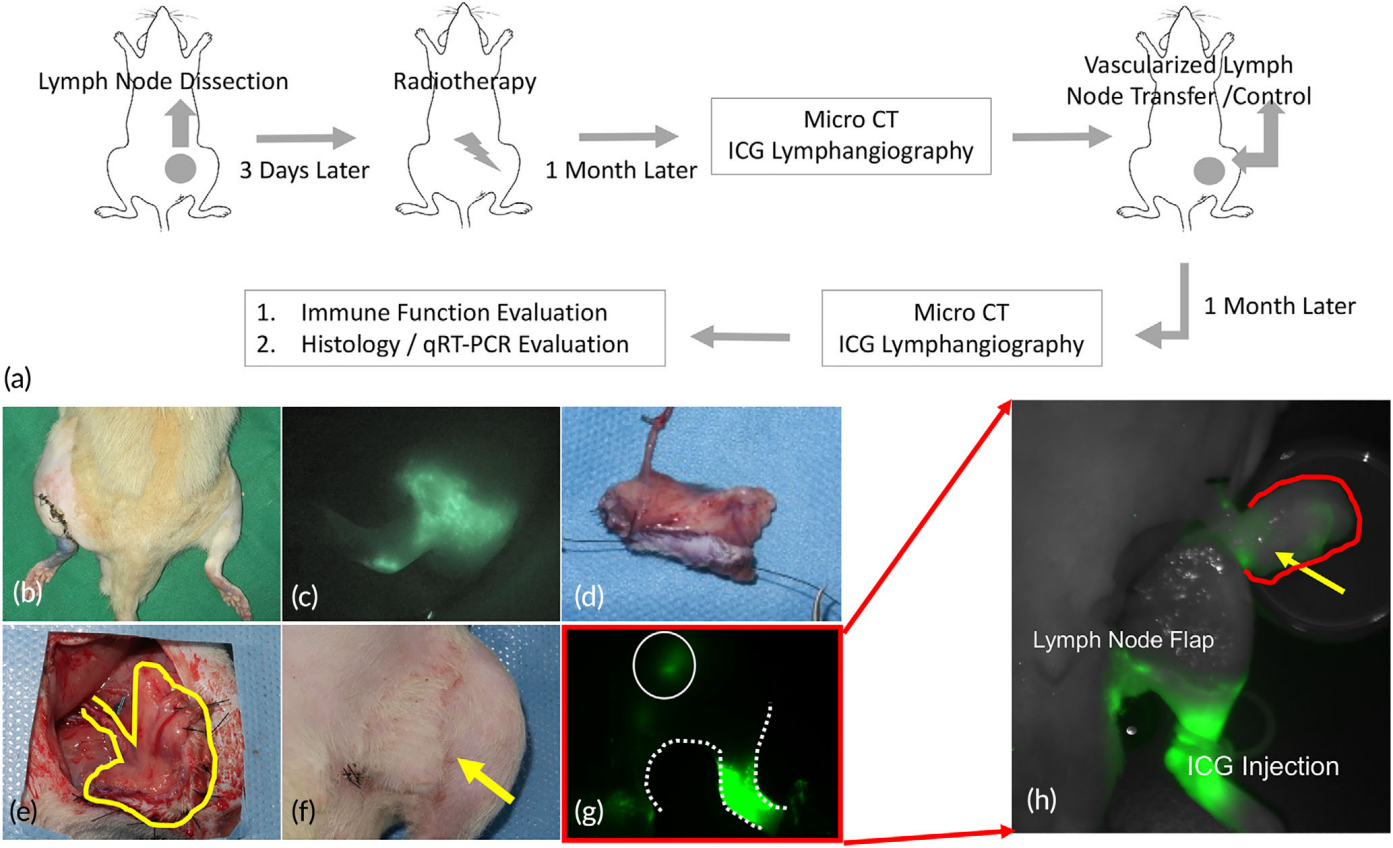
Animal model of vascularized lymph node transfer (VLNT). (a) Diagram of the creation of lymphedema followed by regional radiotherapy 3 days postoperatively. Micro‐computer tomography (CT) and indocyanine green (ICG) lymphangiography were performed to confirm lymphedema 1 month postoperatively. VLNT was performed 1 month after inducing lymphedema. Micro‐CT and ICG lymphangiography were performed 1 month after VLNT. Animals were then sacrificed for immune function and histology evaluation. (b) Representative photo of successfully developed lymphedema with hindlimb swelling. (c) Pattern of dermal backflow type of lymphatic drainage in ICG lymphangiography in rats confirmed the presence of lymphedema. (d) Groin fat pads with skin paddles were harvested as groin lymph node flaps. (e) The recipient site was prepared by gentle dissection of the femoral artery and veins. After vascular preparation, the prepared groin lymph node flap was transferred to the recipient site. (f) After flap inset, a skin paddle was left for perfusion monitoring. (g and h) ICG lymphangiography was performed again before tissue harvest (g), and the transferred lymph node was localized using ICG before tissue harvest and was further confirmed after tissue harvest (h)

**FIGURE 2:**
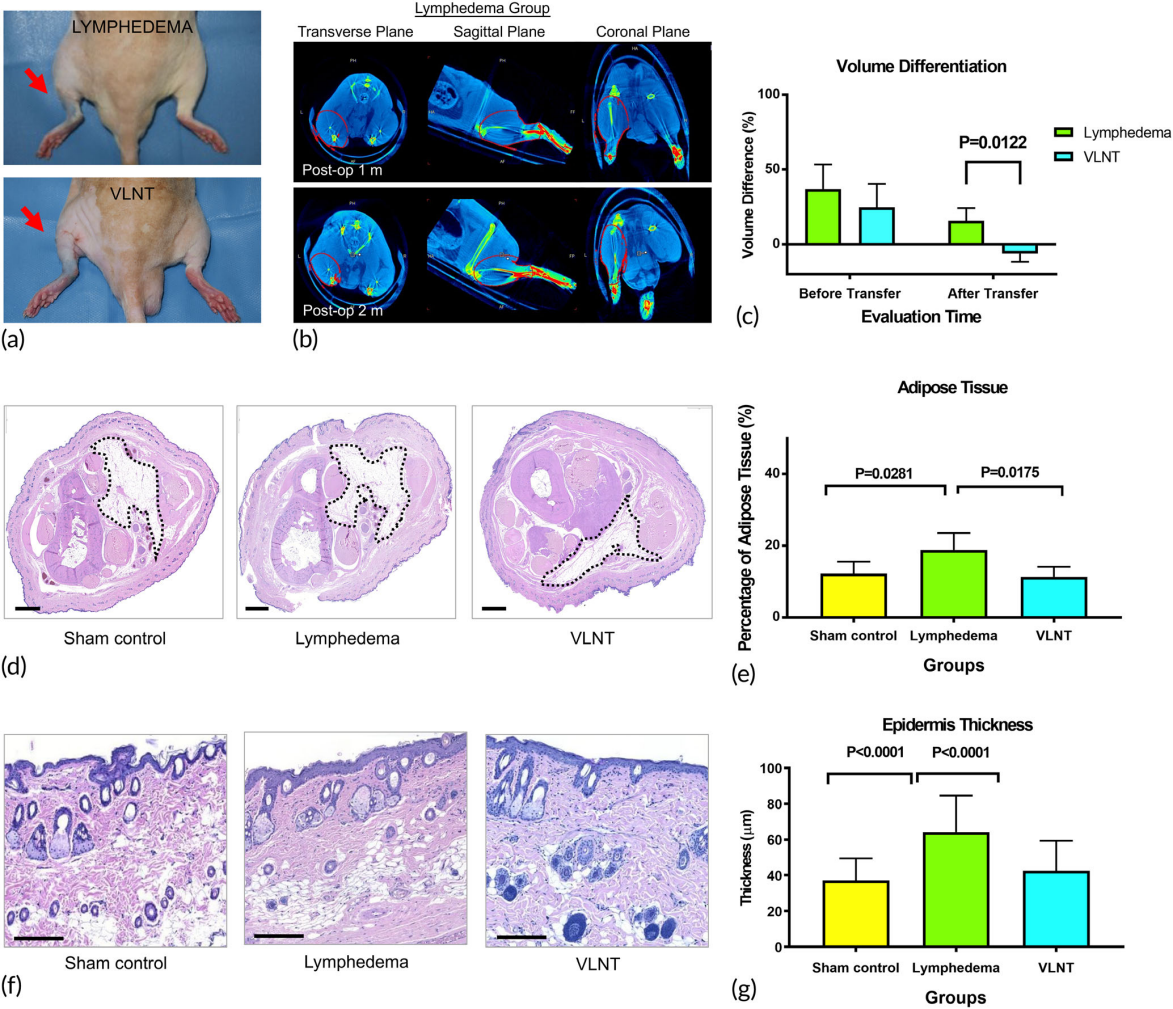
Vascularized lymph node transfer (VLNT) reverses lymphedema as demonstrated by decreased volume and pathologic features. (a) Swelling improvement noted grossly 1 month post‐VLNT. (b) Volume differentiation of the limb of interest was analyzed at 1 and 2 months postoperatively using the micro‐computer tomography reconstructed 3D volume. (c) Quantification of volume differentiation before and after VLNT (*n* = 6). (d) Representative H&E staining of the hindlimb cross‐sections. The dotted circled area indicates fibroadipose tissue; scale bar = 1000 μm. (e) Quantification of the area of fibroadipose tissue (*n* = 6). (f) Representative H&E staining of hindlimb skin, showing epidermal thickness; scale bar = 200 μm. (g) Quantification of epidermal thickness (*n* = 6)

In accordance with clinical experience, VLNT successfully reduced the volume difference between the lymphedema and contralateral healthy hindlimb. After lymphedema creation, the volume differentiation between the hindlimb of interest and the contralateral healthy hind limb was similar in the lymphedema and VLNT groups (24.54 ± 6.35% vs. 30.02 ± 7.28%, *p* = 0.1881). Before vascularized lymph node flap transfer, the dermal back flow pattern of lymphatic draining was confirmed (Figure 1b,c). One month after VLNT, volume differentiation between the hindlimb of interest and the contralateral healthy hind limb was significantly different between the VLNT and lymphedema groups (−6.21 ± 2.20% vs. 9.39 ± 6.68%, *p* = 0.0122). Comparisons within the VLNT group also revealed a significant volume difference before but not 1 month after VLNT (24.54 ± 6.35% vs. −6.21 ± 2.20%, *p* = 0.001) (Figure 2a–c).

### 2.2 VLNT effectively reduced adipose deposition and hyperkeratosis in lymphedema

Along with the improvement of the volume difference, the adipose deposition reduced after VLNT, which echoed clinical scenario. From the cross‐sections of the hindlimbs from the sham surgery, lymphedema, and VLNT groups, the percentage of the area of adipose tissue was significantly lower in the VLNT group than in the lymphedema group (11.30 ± 2.80% vs. 18.81 ± 4.72%, *p* = 0.0175) (Figure 2d,e). Moreover, the thickness of the epidermis was significantly different between the sham and lymphedema groups (37.22 ± 12.27 μm vs. 64.13 ± 20.43 μm, *p* < 0.0001). After VLNT, the thickness of the epidermis was similar between the VLNT group and the sham group (42.48 ± 16.93 μm vs. 37.22 ± 12.27 μm, *p* = 0.0725). Hyperkeratosis with increased thickness of the dermis and epidermis has been considered a hallmark of lymphedema. Similarly, the reduction of the thickness has been considered a positive sign of lymphedema treatment, in both rat tail model and hindlimb model.^22^, ^23^, ^24^ A significant difference also presented between the VLNT and lymphedema groups (42.48 ± 16.93 μm vs. 64.13 ± 20.43 μm, *p* < 0.0001), suggesting that VLNT can successfully convert hyperkeratosis to a normal status (Figure 2f,g).

### 2.3 VLNT effectively reduced T‐cell mediated inflammation

**FIGURE 3:**
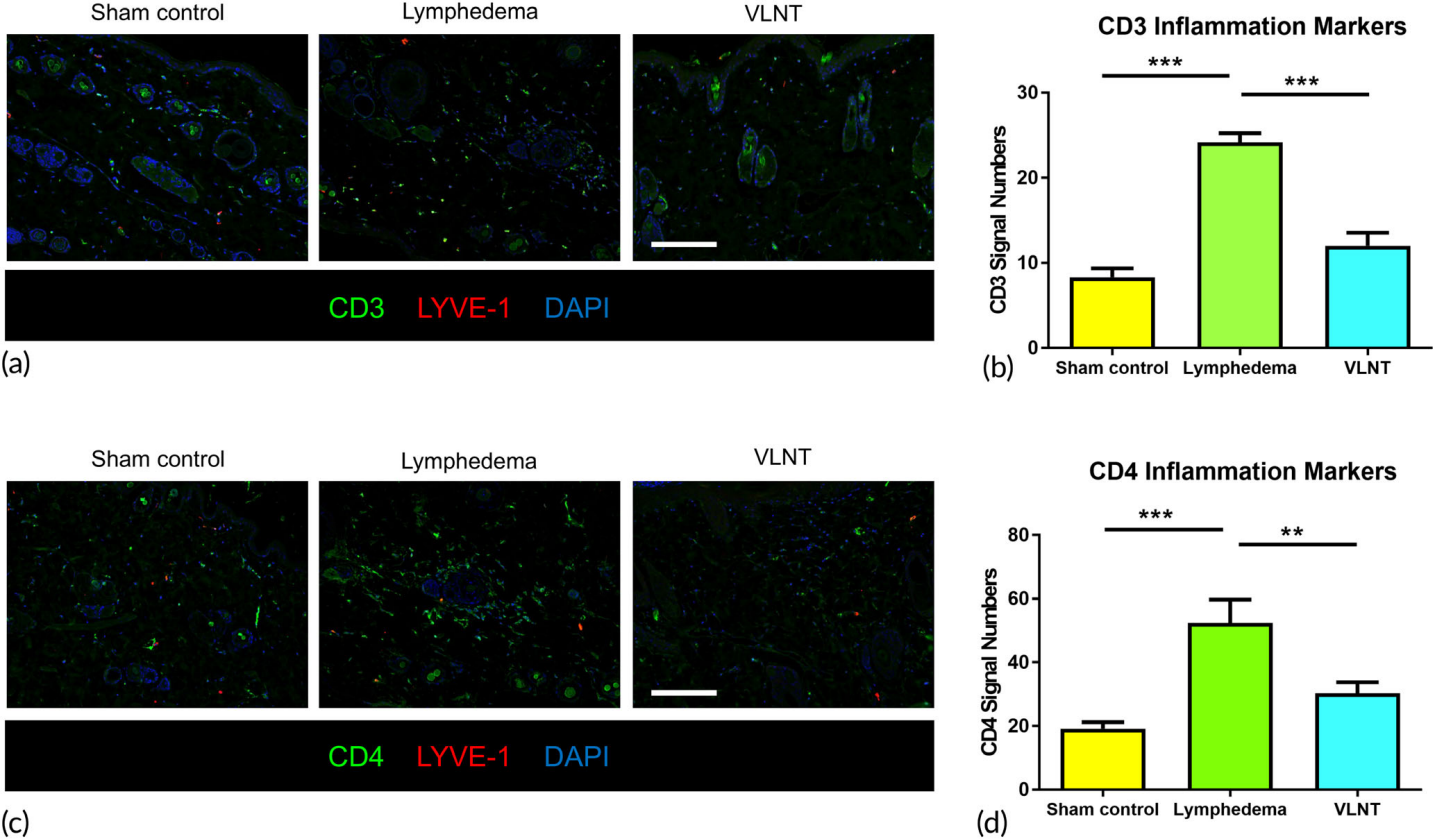
Vascularized lymph node transfer effectively reduced T‐cell mediated inflammation. (a) Representative immunofluorescence images showing CD3+ infiltration in hindlimb skin; scale bar = 50 μm. (b) Quantification of CD3+ T cells in hindlimb skin (*n* = 6). ** indicated *p* < 0.01, *** indicated *p* < 0.001. (c) Representative immunofluorescence images showing CD4+ infiltration in hindlimb skin; scale bar = 50 μm. (d) Quantification of CD4+ T cells in hindlimb skin (*n* = 6)

Lymphedema has been proven to be a T cell‐mediated inflammatory disease.^25^, ^26^, ^27^ Our results revealed more CD3 and CD4 T cell infiltration in the skin from the lymphedema hind limbs than in the skin from the sham control group (CD3: 24.13 ± 4.29 cells/high‐power field [HPF] vs. 8.30 ± 5.63 cells/HPF; CD4: 52.33 ± 28.44 cells/HPF vs. 19.13 ± 11.66 cells/HPF, *p* < 0.0001) and that there was no significant difference between the lymphedema hindlimb receiving VLNT and the skin from the sham control group (CD3: 12.00 ± 5.94 cells/HPF vs. 8.30 ± 5.63 cells/HPF; CD4: 30.33 ± 13.33 cells/HPF vs. 19.13 ± 11.66 cells/HPF, *p* < 0.0001). Reductions in both CD3 and CD4 T cell infiltration were observed in the VLNT group in comparison to the lymphedema group (CD3: 12.00 ± 5.94 vs 24.13 ± 4.2911 cells/HPF; CD4: 30.33 ± 13.33 vs 52.33 ± 28.44 cells/HPF, *p* < 0.0001) (Figure 3).

### 2.4 VLNT effectively promotes lymphangiogenesis

**FIGURE 4:**
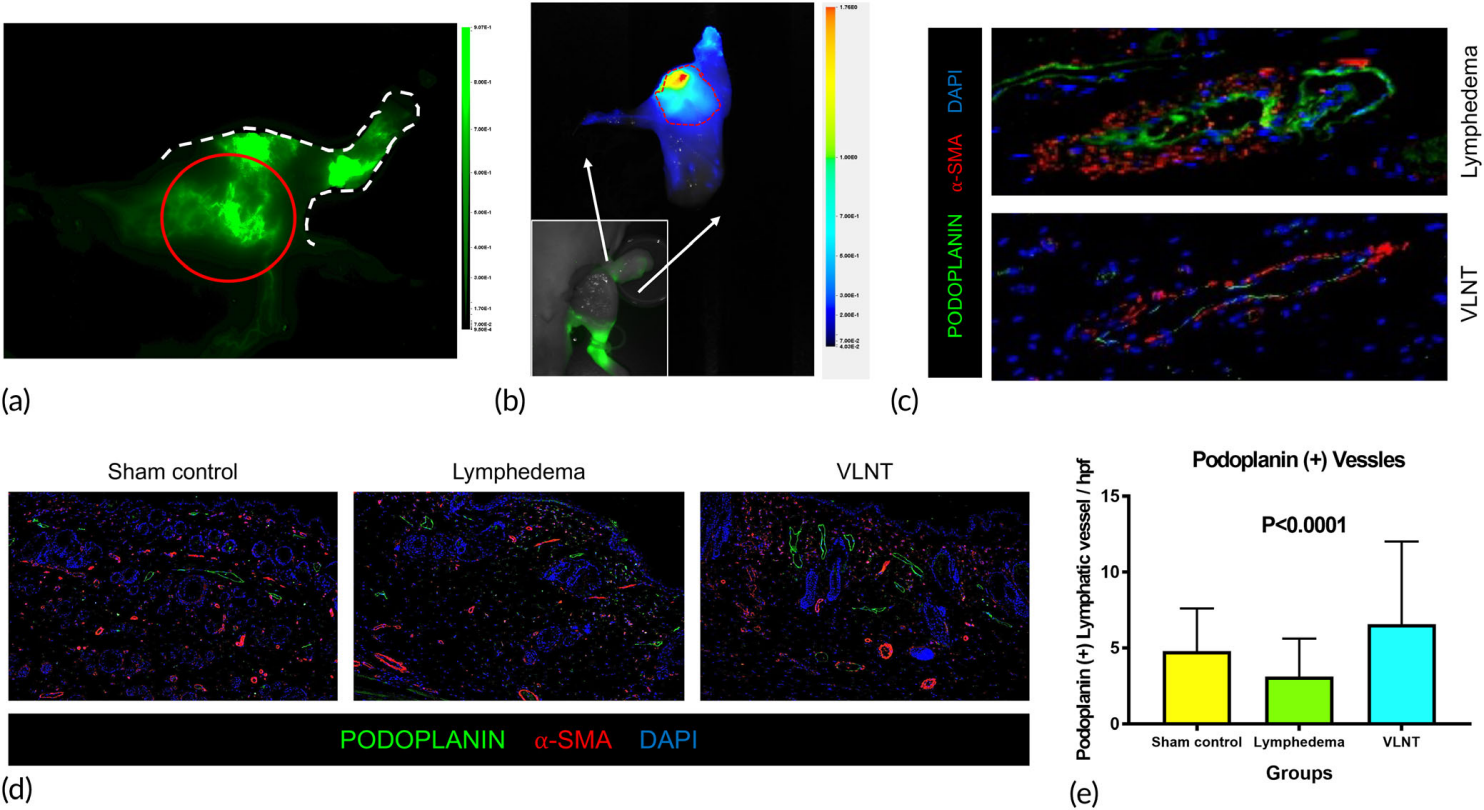
Vascularized lymph node transfer effectively promotes lymphangiogenesis. (a and b) Representative indocyanine green (ICG) lymphangiography showing ICG successfully collected in transferred lymph nodes before (a) and after (b) flap harvest. (c) Representative immunofluorescence images showing collecting lymphatic vessels with α‐smooth muscle actin and podoplanin double‐positive signals (magnification 100×). (d) Representative immunofluorescence images showing the lymphatic architecture in hindlimb skin and subcutaneous tissue (magnification 100×). (e) Quantification of the number of podoplanin+ lymphatic vessels (*n* = 6)

According to the ICG lymphangiography performed before sample harvest, ICG can be identified in the transferred lymph node, suggesting that lymphatic drainage was successfully re‐established after VLNT (Figure 4a,b). Although the dermal back flow remained in the distal hindlimb, lymphatics with a linear pattern presented around the transferred lymph node (red circle in Figure 4a). Collecting lymphatic vessels presenting with double staining of podoplanin and α‐SMA were identified in both the lymphedema and VLNT groups (Figure 4c). However, the α‐SMA was thicker, and the lumen of the collecting lymphatic tissue seemed to have collapsed in the lymphedema group. The collecting lymphatic vessels were grossly healthier with a patent lumen after VLNT. Figure 4d,e reveal that the density of podoplanin‐positive vessels was significantly higher in the VLNT group, suggesting a higher density of lymphatic vessels in the VLTN group after lymphangiogenesis. (podoplanin[+] lymphatic vessels: Sham: 4.8 ± 2.8/HPF, Lymphedema: 3.1 ± 2.5/HPF, VLNT: 6.8 ± 5.4/HPF, *p* < 0.0001.)

### 2.5 VLNT successfully maintained local immune cell trafficking and systemic immune response

**FIGURE 5:**
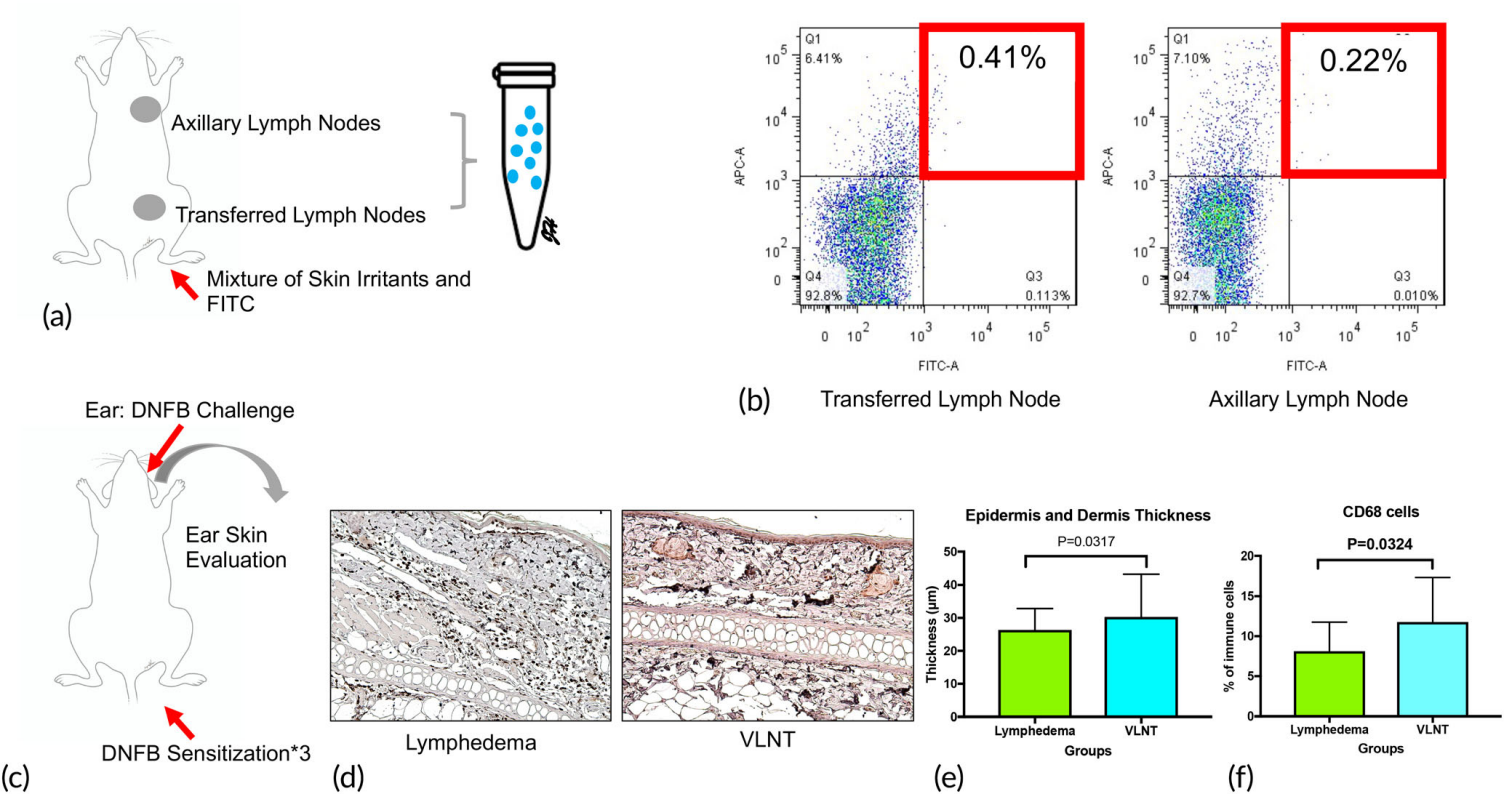
Vascularized lymph node transfer successfully maintained local immune cell trafficking and systemic immune responses. (a) Schematic diagram of ipsilateral axillary and transferred lymph nodes harvested for analysis 18 h after fluorescein isothiocyanate (FITC)‐conjugated skin irritants were painted on the limbs. (b) Representative FITC plots of CD11b+ and FITC double‐positive macrophages isolated from both axillary and transferred lymph nodes. (c) Schematic diagram showing ear skin harvested after dinitrofluorobenzene (DNFB) challenge. Three DNFB sensitizations were applied on the skin of the lymph node‐transferred limb. (d) Representative immunohistochemistry (IHC) images of ear skin after DNFB challenge (magnification 200×). (e) Quantification of epidermal thickness by IHC (*n* = 6). (f) Quantification percentage of CD68+ cell occupied area by IHC (*n* = 6)

To understand whether the newly developed lymphatic channels are effective in transporting immune cells, dendritic cell trafficking was assessed using the fluorescein isothiocyanate (FITC) painting technique. The hindlimb of interest was painted with a mixture of FITC and skin irritants, and the transferred lymph nodes and ipsilateral axillary lymph nodes were harvested 18 h later for investigation (Figure 5a). CD11b and FIFC double‐positive macrophages were identified by flow cytometry. They accounted for 0.41 ± 0.16% and 0.22 ± 0.03% of the transferred lymph nodes and axillary lymph nodes, respectively (*p* = 0.018) (Figure 5b). This suggests that the re‐established lymphatic channels are capable of transporting macrophages from the distal region via the transferred lymph node and then to the lymph node of the next stream.

After confirmation of immune cell trafficking, we next investigated whether the systemic immune response could be re‐established. Three doses of dinitrofluorobenzene (DNFB) sensitization were applied to the hind limb of interest. DNFB challenge was applied to the ear, and the ear skin was then harvested for analysis. Sections from the DNFB‐challenged ear skin were harvested for immunohistochemistry (IHC). Increased thickness of the epidermis and dermis was observed in the ear skin of the VLNT group (30.32 ± 12.94% vs. 26.36 ± 6.48%, *p* < 0.05), accompanied by a higher percentage of CD68+ cell infiltration (15.09 ± 1.51% vs. 10.97 ± 0.68%, *p* < 0.05). This suggested that more inflammatory reactions were recalled in the VLNT‐treated group than in the lymphedema group (Figure 5d–f).

### 2.6 The transferred lymph nodes maintained a normal structure

**FIGURE 6:**
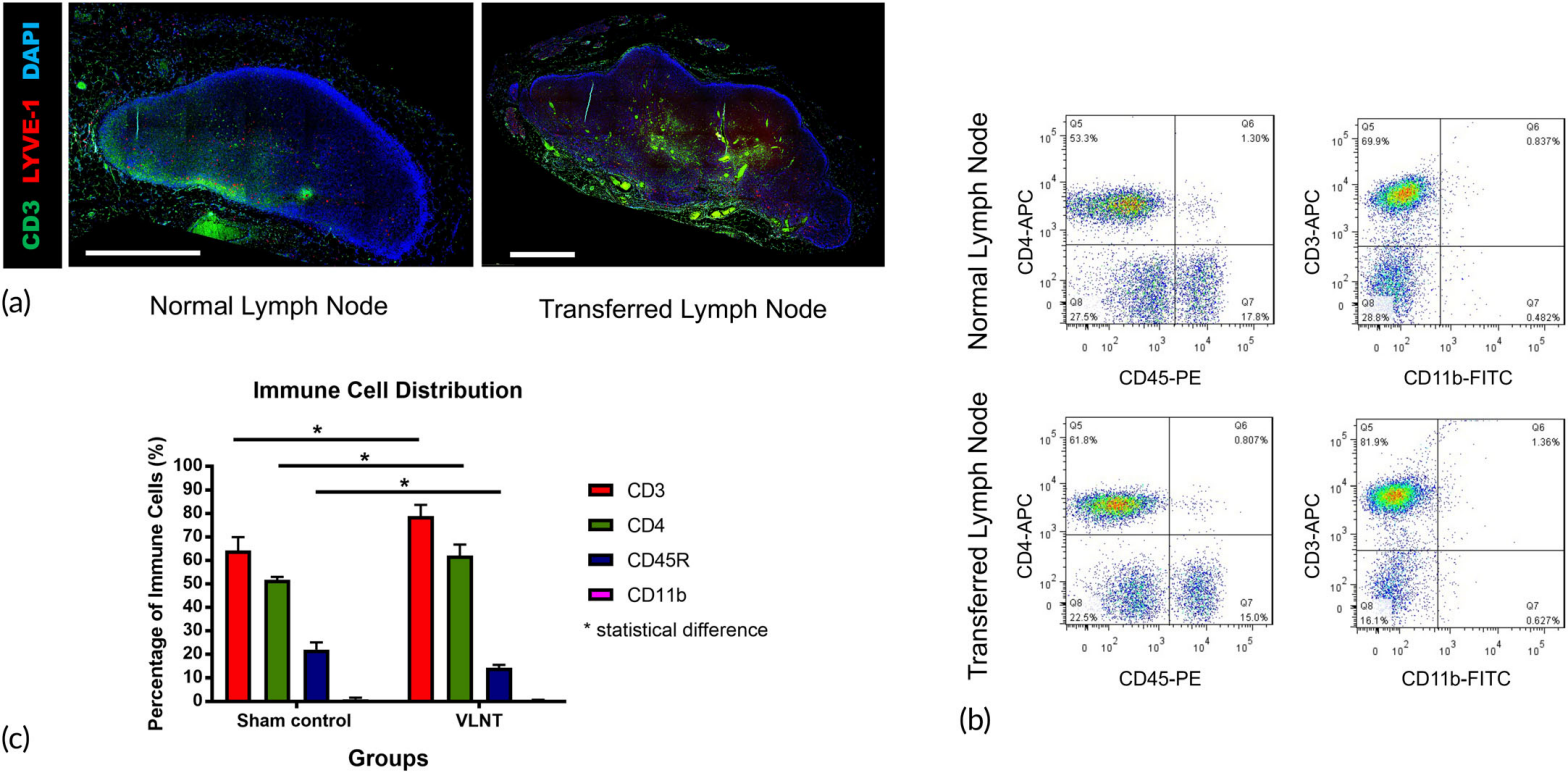
Transferred lymph nodes maintained a normal structure. (a) Representative immunofluorescence staining of normal and transferred lymph node architecture; scale bar = 1000 μm. (b and c) FITC plot (b) and quantification (c) of lymphocyte subsets inside normal and transferred lymph nodes (*n* = 6)

The microscopic morphology of the transferred lymph nodes confirmed that they maintained a basic anatomical structure with T cells located in the paracortical area (Figure 6a). Flow cytometry was performed to identify CD3, CD4, CD45R, and CD11b. While all types of immune cells were identified, the distribution ratio was different between the transferred and normal lymph nodes. A significantly higher percentage of CD3+ and CD4+ T cells was present in the transferred lymph nodes than in the normal lymph nodes (CD3: 78.95 ± 4.16% vs. 64.20 ± 5.00%, *p* < 0.01; CD4: 62.00 ± 4.15% vs. 51.73 ± 1.04%, *p* < 0.01); at the same time, the percentage of CD45R+ cells was lower in the transferred lymph nodes (14.18 ± 1.15% vs. 21.92 ± 2.72%, *p* < 0.01) (Figure 6b,c).

### 2.7 Gene expression indicating lymphatic regeneration after VLNT

**FIGURE 7:**
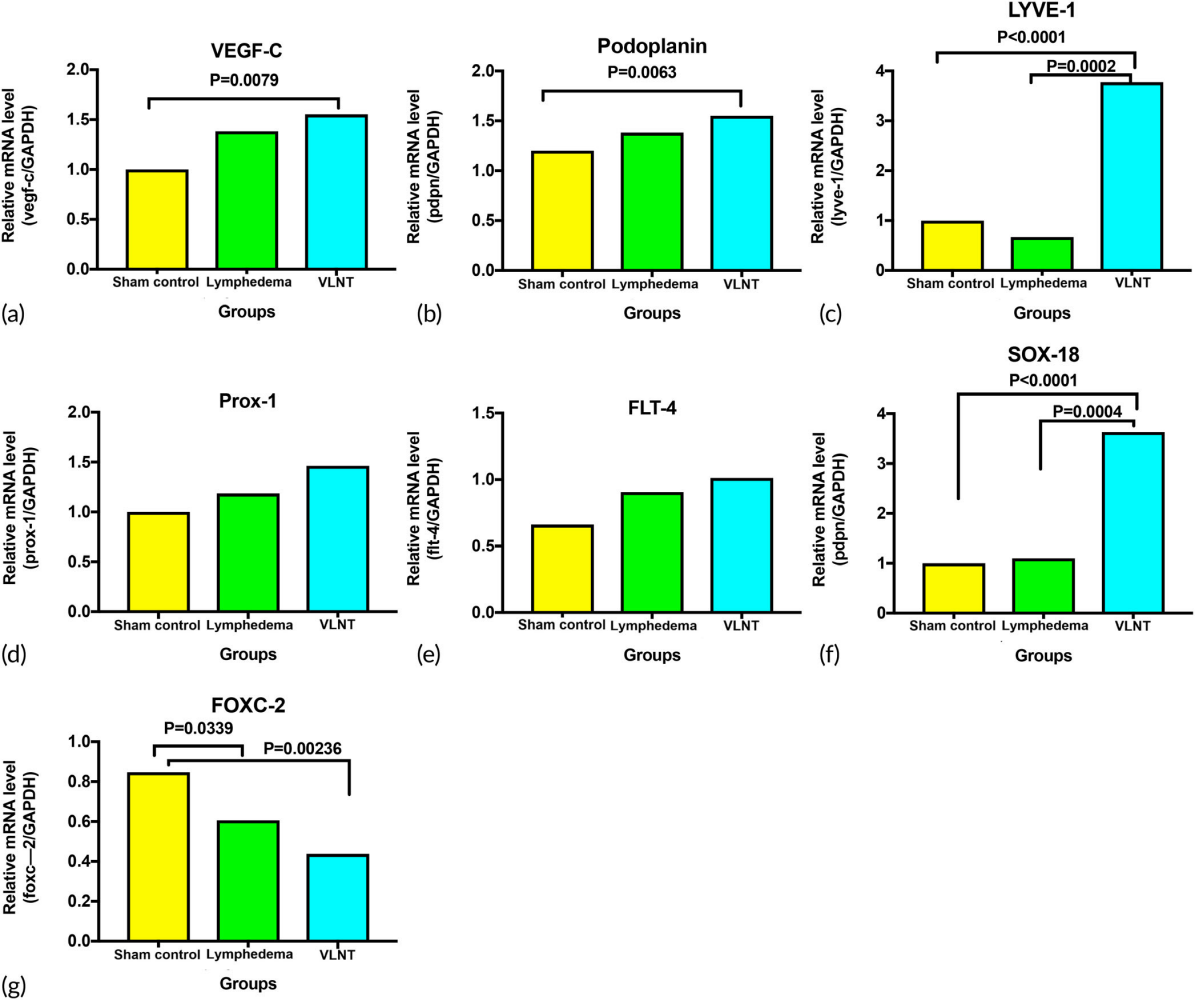
Gene expression indicating lymphatic regeneration after vascularized lymph node transfer. Relative mRNA levels of the following genes in the harvested skin and subcutaneous tissue: (a) VEGF‐C, (b) podoplanin, (c) LYVE‐1, (d) Prox‐1, (e) FLT4/VEGFR3, (f) Sox‐18, and (g) FOXC‐2 (*n* = 6)

Skin and subcutaneous tissue were harvested from the lymphedema‐ and VLNT‐treated groups for lymphangiogenesis‐related genetic expression of analysis using real‐time quantitative reverse transcription polymerase chain reaction (qRT‐PCR). The sham surgery group was taken as the control. Genes mediating lymphangiogenesis and patterning were screened. Upregulation of vascular endothelial growth factor C (VEGF‐C) and podoplanin was observed in the VLNT and lymphedema groups in comparison to the control group. Interestingly, lymphatic vessel endothelial hyaluronan receptor‐1 (LYVE‐1) and Sox‐18 upregulation were only observed in the VLNT group. The expression levels of other genes, including VEGFR3 (FLT‐4) and Prox‐1, were similar among all three groups. Downregulation of FOXC2 was noted in both the VLNT and lymphedema groups (Figure 7).

## 3 DISCUSSION

Considering the limitations of understanding recovery mechanism of lymphedema in clinical cases after VLNT, we applied a surgical animal model of lymphedema followed by VLNT to investigate its effect.^28^ Our results revealed that VLNT treated lymphedema with recovery of volume reduction in micro‐CT, lymphatic transportation in ICG lymphangiography, and reductions in the histological hallmarks of lymphedema. Furthermore, the maintenance of immunity can be identified after VLNT, including dendritic cell trafficking and systemic immune reactions. Re‐establishment of the lymphatic channel is the main purpose of physiological treatment of lymphedema. Our study provides evidence that lymphangiogenesis can be promoted and lymphatic channel can be re‐established after VLNT.

ICG lymphangiography revealed a dermal back flow in the distal hindlimb and more lymphatics with a linear pattern can be seen around the transferred lymph node, suggesting that there was more lymphangiogenesis around the transferred lymph node than the distal hindlimb (Figure 4a). Besides, dendritic cell trafficking can be seen from the distal hindlimb to the transferred groin lymph node, and a few of the cells are subsequently transported to the axillary lymph nodes (Figure 5).

For the mechanism of lymphedema recovery from VLNT, drainage via natural lymphaticovenous connections inside the lymph node has been suggested.^29^ However, the route for dendritic cell trafficking provided evidence that the re‐establishment of lymphatic drainage seemed to be more than just from the natural lymphaticovenous connections inside the lymph nodes. Our results suggested that VLNT promoted lymphangiogenesis, with more podoplanin(+) lymphatic vessels present in the VLNT group (Figure 4d,e). Similar evidence can be seen from the gene expression analysis of several of the key factors of lymphangiogenesis. There was a tendency of more but not significantly increases in the expression of VEGF‐C, LYVE‐1, podoplanin, PROX‐1 and FLT4 in the lymphedema group in comparison to the normal group, suggesting that lymphangiogenesis occurs when lymphatic injury, tissue injury or lymphedema presents. However, the VLNT presented a significant increase relative to the normal tissue, suggesting it is a more effective trigger of lymphangiogenesis. Significant upregulation of the VEGF‐C, podoplanin, LYVE‐1, and SOX‐18 genes and a tendency of more FLT‐1 and Prox‐1 presentation also provided evidence of lymphangiogenesis from the initial stage of lymphatic development to the later stage after VLNT (Figure 7). Prox‐1 and LYVE‐1 are key features of the initial development of the lymphatic vasculature, and VEGF‐C and thus FLT‐4, as its receptor, are essential for development.^30^, ^31^ Sox‐18 and VEGF‐C have a strong interaction in the early phase of lymphatic development in animal studies.^32^ Our results also highlighted the increased presence of Sox‐18 in the VLNT group, which echoed lymphangiogenesis for capillary lymphatics. Interestingly, FOXC‐2, which is not a key factor in the development of capillary lymphatics but is crucial for patterning lymphatics into collecting lymphatic vessels, was downregulated in our results. This may explain why lymphangiogenesis following VLNT was mainly associated with capillary lymphatics.

In addition to drainage, frequent infection is one prominent feature symptom of lymphedema patients beyond the presence of extremity swelling and fibrosis. The recovery of immune defense should be considered equally important to successful fluid transport, including antigen presentation and effective immune response by naïve T cell differentiation.^33^, ^34^

After confirming the improvements in characteristics of swelling and lymphedema, we next investigated immune function after VLNT. Our results showed that dendritic cell trafficking and the systemic immune response can both be restored after VLNT. A few of the dendritic cells can further travel to the axillary lymph nodes, which are the next stream of lymph nodes to the groin lymph nodes, suggesting the connection from the transferred lymph node to the next ones.

The challenge of DNFB, followed by DNFB stimulation from remote areas (ears) other than the hindlimb, provides evidence of the recovery of systemic immune reactions. Similar results have been reported earlier in non VLNT in a mouse model.^35^ The present study further confirmed the restoration of immune function in a VLNT model, providing evidence of immune recovery in a clinical scenario and that patients receiving vascularized lymph nodes present with less cellulitis than patients who have lymphedema without treatment.^36^

Interestingly, while the transferred vascularized lymph node maintained its basic structure with a typical distribution of immune cells, the flow cytometry results confirmed that the numbers of CD3(+) and CD4(+) T cells were significantly higher in the transferred lymph nodes than in the normal/nonoperated lymph nodes. Conversely, CD45R(+) B cells were more abundant in the normal lymph nodes (Figure 6). The exact reason for this remains unknown. It can likely be from the surgery itself or because of the recipient site. Lymphedema is a T cell‐mediated inflammatory disease.^25^, ^26^, ^27^ Additional study will be required to investigate this in depth.

## 4 METHODS

This study was completed using a rat animal model. Hind limb lymphedema was created surgically by removing the groin and popliteal lymph nodes followed by postoperative radiation to mimic the clinical scenario. The presence of lymphedema was confirmed with microcomputer tomography (micro‐CT) and indocyanine green (ICG) lymphangiography 1 month after radiotherapy, identifying the presence of volume differences and confirmation of the dermal back flow. The animals were grouped into VLNT and control lymphedema groups. The final evaluation was performed another month later with a volume measurement using micro‐CT and ICG lymphangiography. Immune recovery was surveyed. The animals were then euthanized, and histological evaluation was completed (Figure 1a). In addition, animals receiving sham surgery with an oblique incision over the groin area with primary closure were prepared as the control group (*n* = 6 in each group).

### 4.1 Animals

All animal procedures were approved by the Institutional Animal Care and Use Committee (IACUC number: 20151804) of Chang Gung Memorial Hospital and were performed in accordance with the institution's animal research guidelines. Male Lewis rats weighing 400 to 450 g were obtained from BioLASCO Taiwan Co., Ltd. The animal surgeries were conducted under anesthesia using 2.5% isoflurane inhalation via a nose cone. After confirmation of the depth of anesthesia, the skin was prepared by removing the fur from an area 150% larger than the incision area. The skin was then prepared in an aseptic manner.

### 4.2 Creation of unilateral hind limb lymphedema

After induction of anesthesia, 0.1 ml of 10% Evans Blue (Sigma‐Aldrich) was injected subcutaneously into the dorsum of the paw. An oblique inguinal incision was made, and inguinal and popliteal lymph nodes along with the interposed lymphatic vessels were subsequently identified and completely excised with inclusion of the surrounding fat tissue. The surgical sites were irradiated with a single dose of 20 Gy radiation (Varian 2100 EX Linear Accelerator, Medical Imaging Resources, Inc.) 3 days after the surgery. The dimensions of the radiation field were 4.5 × 5 cm with a depth of 0.2 cm. Subsequent lymphedema formation was quantitatively assessed by micro‐CT imaging (Figure 2b) and further confirmed using ICG lymphangiography (Figure 1c).

### 4.3 Micro‐CT imaging

The volume of the hind limbs was measured by using Nano‐SPECT/CT (NanoSPECT/CT, Bioscan) with the animals in a supine position 1 month after radiotherapy. The rats were under general anesthesia as previously described.^20^, ^21^ The scan was taken for 15 min, and the images were reconstructed with filtered back‐projection into a 3D‐image volume with pixels sized 0.2 mm in both the transverse and axial directions. All images were saved in DICOM format and later analyzed with PMOD software (PMOD Technologies Ltd.).

The volume differentiation was determined by the following equation:

(Volume of the hind limb of interest − Volume of the contralateral healthy hind limb)/Volume of the contralateral healthy hind limb × 100%.

A volume differentiation greater than 5% was considered swelling from lymphedema.

### 4.4 Harvesting the vascularized lymph node flap

Vascularized groin lymph node flaps were harvested from the donor rats' inguinal areas.^37^, ^38^ Briefly, an incision was made along the groin area. Subcutaneous dissection was carried out carefully, and all of the subcutaneous fat pads were preserved. The fat pad, including the lymph nodes, was then dissected carefully along with its supply vessels, mainly the superficial epigastric artery and vein. The vessels were further dissected to include part of the femoral artery and vein to facilitate microvascular anastomosis (Figure 1d. The donor wounds were then closed. Postoperative pain medication was given immediately after the surgery.

### 4.5 Transferring a vascularized lymph node flap

An incision was made in the groin area of the hind limb of the lymphedema rats. The femoral artery and vein were explored after layer‐by‐layer dissection. The vessels were prepared carefully by dissection of a sufficient length for vascular anastomosis. The adventitia was removed as much as possible. After vascular preparation, the prepared groin lymph node flap was transferred to the recipient site, and anastomosis was performed in an end‐to‐end manner with 11–0 nylon. The vascularized lymph node flap was inset into the recipient site and the wound closed (Figure 1e,f).

### 4.6 ICG lymphography

The use of near‐infrared images with ICG injection have been developed for lymphatic identification, lymphedema evaluation and intraoperative guidance for lymphatic surgeries.^39^ Briefly, ICG was injected into the foot paw of the hind limb of interest. The images were taken 30 min after the injection (to allow ICG transport) by a Pearl Impulse Small Animal Imaging System (LI‐COR Biotechnology). Lymphangiography was performed 1 month after the lymphedema creation surgery to confirm the lymphedema status (Figure 1c) and repeated after VLNT to confirm lymphatic drainage (Figure 1g,h).

### 4.7 Histological examination and immunofluorescence analysis

For immunofluorescence (IF) analysis, the samples were harvested from the skin of the hind limb of interest at a size of 2 × 2 cm. Samples were then fixed with 10% formaldehyde for 12 to 14 days at room temperature, embedded in paraffin and sectioned for H&E and IF staining. The slides were rehydrated, and heat‐induced antigen retrieval was performed using 95°C citric acid (S2369, Dako). Nonspecific binding was blocked with antibody diluent (S3022, Dako) for 30 min. Samples were incubated with the following primary antibodies overnight at 4°C: anti‐LYVE‐1 (1:200, ab10278, Abcam), anti‐collagen I (1:200, ab34710, Abcam), anti‐CD3 (1:200, ab699, Abcam), anti‐CD4 (1:200, ab133616, Abcam), anti‐CD45 (1:200, ab10558, Abcam), and anti‐alpha smooth muscle actin (α‐SMA) (1:200, ab21027, Abcam) and anti‐podoplanin (1:200, 14–5381‐85, Invitrogen). IF staining was performed using the following secondary antibodies: Alexa Fluor® 488‐conjugated donkey anti‐rabbit IgG (1:500, ab150061, Abcam), Alexa Fluor® 488‐conjugated goat anti‐mouse IgG (1:500, ab150117, Abcam), Alexa Fluor® 594‐conjugated goat anti‐mouse IgG (1:500, ab150120, Abcam) and Alexa Fluor® 594‐conjugated goat anti‐rabbit IgG (1:500, ab150080, Abcam). Nuclear staining was performed using DAPI.

### 4.8 DNFB sensitization and IHC

The rats were sensitized to 2,4‐dinitrofluorobenzene by painting the foot paw and distal of the lymphedema hindlimb with 500 μl 0.25% DNFB (Sigma Aldrich, Merck) in acetone/olive oil (4:1, Sigma Aldrich) 5 and 6 days before sacrifice. Their ears were challenged with 100 μl DNFB 1 day before sacrifice. The ears were harvested to evaluate inflammatory reactions.

The inflammation of the DNFB‐challenged ear was analyzed by CD68 staining. IHC studies were performed using the Dako Real™ EnVision™ Detection System Peroxidase/DAB+, Rabbit/Mouse (Dako).

The sections were dewaxed in xylene and rehydrated in graded alcohol. Antigen retrieval was performed by heating in citrate buffer solution at 95°C for 20 min. Sections were blocked with 3% hydrogen peroxide for 10 min. Immuno‐enhancer buffer (ACE Biolabs) was used for protein blocking and as the primary antibody diluent. The anti‐CD68 antibody (ab955, 1:200, Abcam) was applied for 2 h of incubation at room temperature. Super Enhancer was applied as a second antibody for 30 min at room temperature. Between these steps, the slides were washed with PBS with Tween® 20 solution. Then, DAB was applied for color development. A positive reaction resulted in brown staining, followed by counterstaining with hematoxylin solution (Dako). The expression area was counted by the Image‐Pro Premier program (Media Cybernetics) and normalized to the whole area of the section.

### 4.9 FITC trafficking

FITC trafficking was designed to investigate the recovery of dendritic cell trafficking after VLNT. Briefly, 1% FITC (Sigma‐Aldrich) in 1 ml acetone mix with olive oil (1:1) was painted on the hind limb of interest after VLNT. The transferred lymph node and the ipsilateral axillary lymph node were harvested 18 h later, and flow cytometry was performed to count the trafficked CD11b and FITC double‐positive cells.

### 4.10 Flow cytometry

The lymph nodes were removed into Petri dishes containing 5 ml PBS, rubbed, and broken to make cell suspensions. The suspension was centrifuged at 1500 rpm for 5 min. The supernatant was discarded, and 2 ml ammonium‐chloride‐potassium (ACK) lysis buffer was added and incubated at room temperature for 1 min. Then, 2 ml PBS was added to neutralize the ACK lysing buffer. The suspension was centrifuged at 1500 rpm for 5 min. The supernatant was discarded, and then 2 ml PBS was added to resuspend the cells. The suspension was then filtered through a 100 μm strainer (Falcon, Corning) to obtain a single cell suspension. The cells were stained with fluorochrome‐labeled anti‐rat monoclonal antibodies against CD3, CD4, CD11b or CD45R (BD Bioscience) to identify T cells, macrophages and B cells. The cells were analyzed by two‐color flow cytometry using a BD FITC Canto II (BD Bioscience) and FlowJo software (FlowJo). The cells were gated to exclude residual tissue debris and nonviable cells, and sample data were collected of 20,000 cells. For evaluation of immune cell trafficking, similar procedures were performed to identify the CD11b and FITC double‐positive cells.

### 4.11 Real‐time qRT‐PCR

The harvested skin and subcutaneous tissue were overlaid with TRIzol (Invitrogen) and stored. RNA was isolated using the RNeasy kit (QIAGEN) according to the manufacturer's protocol. One microgram of RNA was reverse transcribed with a High‐Capacity cDNA Reverse Transcription kit (Applied Biosystems). FastStart SYBR Green master mix (Roche, Basel, Switzerland) was used for the quantitative PCR assays and analyzed with a StepOnePlus Real‐Time PCR System (Applied Biosystems). All gene expression values were normalized to the GAPDH (mouse) levels for each sample. Table 1 shows the primers used in the qRT‐PCR.

**TABLE 1:** Primer sequences for qRT‐PCR

Primer	Forward sequence	Reverse sequence
FLT‐4	GACTAAGGCTCCAGGTTCCTCC	TCCCGCTGTCTGTTTGGTTAT
Podoplanin	GCTTCATTGGAGGGATCATCAT	AGACCTGGGTTCACCATGTCA
LYVE‐1	CGAGGCACCCAGTCCATG	CTGCATTTCTGCCCACGAC
PROX‐1	AGAGCCCTCAACATGCACTACA	CTTCCAGGAAGGATCAACATCTTT
VEGF‐C	CGTCTACAGATGTGGGGGTTG	CTGGTTTGGGGCCTTGAGA
FoxC2	CTACCAGTTCATCATGGACCGTT	CCTTCACGAAGCACTCGTTG
Sox‐18	TGAACGCCTTTATGGTGTGG	TCTCCGCCGTGTTCAGCT
GAPDH	CAAGT TCAAC GGCAC AGTCAAG	AGCAC CAGCA TCACC CCAT

Abbreviation: qRT‐PCR, quantitative reverse transcription polymerase chain reaction.

### 4.12 Statistics

Statistical analysis was performed using GraphPad Prism software (GraphPad Software). Comparisons were performed using Student's t‐test or one‐way ANOVA, depending on the grouping, for comparison. A *p*‐value less than 0.05 was considered to indicate a significant difference.

## 5 CONCLUSIONS

In summary, this study provides evidence at the histological and molecular levels to confirm the effectiveness of VLNT in treating lymphedema, which is not possible to collect from clinical cases. More importantly, our study showed that the transferred vascularized lymph node can successfully restore immune function by providing adequate drainage function and restore the adaptive immune system. These findings provide valuable information on the clinical application of VLNT. It also provides fundamental knowledge to continue further lymphatic research.

## CONFLICT OF INTEREST

The authors declared no conflict of interest. The funder of this study had no role in the design, execution, interpretation, or writing of the study.

## AUTHOR CONTRIBUTIONS

**Ahmet Hamdi Sakarya:** Conceptualization (equal); investigation (equal); methodology (equal); writing – original draft (equal). **Chi‐Wei Huang:** Conceptualization (equal); formal analysis (equal); visualization (equal); writing – original draft (equal). **Chin‐Yu Yang:** Formal analysis (equal); investigation (equal). **Hui‐Yi Hsiao:** Investigation (supporting); writing – original draft (supporting); writing – review and editing (equal). **Frank Chun‐Shin Chang:** Conceptualization (supporting); writing – review and editing (equal). **Jung‐ju Huang:** Conceptualization (lead); data curation (lead); formal analysis (equal); funding acquisition (lead); methodology (equal); supervision (lead); writing – original draft (supporting); writing – review and editing (lead).

## INFORMED CONSENT STATEMENT

Not applicable.

## Data Availability

Data available on request due to privacy/ethical restrictions
